# High blood pressure, antihypertensive medication and lung function in a general adult population

**DOI:** 10.1186/1465-9921-12-50

**Published:** 2011-04-21

**Authors:** Eva Schnabel, Stefan Karrasch, Holger Schulz, Sven Gläser, Christa Meisinger, Margit Heier, Annette Peters, H-Erich Wichmann, Jürgen Behr, Rudolf M Huber, Joachim Heinrich

**Affiliations:** 1 Helmholtz Zentrum München, German Research Center for Environmental Health, Institute of Epidemiology, Neuherberg, Germany; 2 Ludwig-Maximilians-University Munich, Dr. von Hauner Children's Hospital, Munich, Germany; 3 Helmholtz Zentrum München, Institute of Lung Biology and Disease, Munich, Germany; 4 Ludwig-Maximilians-University Munich, Institute and Outpatient Clinic for Occupational, Social and Environmental Medicine, Munich, Germany; 5 Comprehensive Pneumology Center, University Hospital of the Ludwig Maximilians University Munich, Asklepios Hospital Gauting and Helmholtz Zentrum München, Munich, Germany; 6 Department of Internal Medicine B - Cardiology, Intensive Care, Pulmonary Medicine and Infectious Diseases, University of Greifswald, Greifswald, Germany; 7 Central Hospital of Augsburg, MONICA/KORA Myocardial Infarction Registry, Augsburg, Germany; 8 Ludwig-Maximilians-University, Institute of Medical Data Management, Biometrics and Epidemiology, Munich, Germany; 9 Ludwig-Maximilians-University, Division of Pulmonary Diseases, Department of Internal Medicine I, Grosshadern, Munich, Germany; 10 Ludwig-Maximilians-University, Division of Respiratory Medicine, Department of Medicine, Innenstadt, Munich, Germany; 11 Helmholtz Zentrum München, German Research Center for Environmental Health, Institute of Epidemiology II, Neuherberg, Germany

## Abstract

**Background:**

Several studies showed that blood pressure and lung function are associated. Additionally, a potential effect of antihypertensive medication, especially beta-blockers, on lung function has been discussed. However, side effects of beta-blockers have been investigated mainly in patients with already reduced lung function. Thus, aim of this analysis is to determine whether hypertension and antihypertensive medication have an adverse effect on lung function in a general adult population.

**Methods:**

Within the population-based KORA F4 study 1319 adults aged 40-65 years performed lung function tests and blood pressure measurements. Additionally, information on anthropometric measurements, medical history and use of antihypertensive medication was available. Multivariable regression models were applied to study the association between blood pressure, antihypertensive medication and lung function.

**Results:**

High blood pressure as well as antihypertensive medication were associated with lower forced expiratory volume in one second (p = 0.02 respectively p = 0.05; R^2^: 0.65) and forced vital capacity values (p = 0.01 respectively p = 0.05, R^2^: 0.73). Furthermore, a detailed analysis of antihypertensive medication pointed out that only the use of beta-blockers was associated with reduced lung function, whereas other antihypertensive medication had no effect on lung function. The adverse effect of beta-blockers was significant for forced vital capacity (p = 0.04; R^2^: 0.65), while the association with forced expiratory volume in one second showed a trend toward significance (p = 0.07; R^2^: 0.73). In the same model high blood pressure was associated with reduced forced vital capacity (p = 0.01) and forced expiratory volume in one second (p = 0.03) values, too.

**Conclusion:**

Our analysis indicates that both high blood pressure and the use of beta-blockers, but not the use of other antihypertensive medication, are associated with reduced lung function in a general adult population.

## Background

Hypertension is an increasingly important public health challenge worldwide and it is one of the major causes for morbidity and mortality [1]. Thus, the National High Blood Pressure Education Program reports that the global burden of hypertension is approximately 1 billion individuals and that more than 7 million deaths per year may be attributable to hypertension [2].

Moreover, hypertension has been linked to multiple other diseases including cardiac, cerebrovascular, renal and eye diseases [3]. Beside the well-established association between hypertension and vascular comorbidities, several studies showed that blood pressure and lung function are associated [4-9]. It could be demonstrated that higher forced vital capacity (FVC) is a negative predictor of developing hypertension [7,8]. Moreover, some studies found an association between reduced pulmonary function, including both low FVC and low forced expiratory volume in one second (FEV_1_), and hypertension [5,9,6].

Furthermore, there are a number of publications discussing the controversial effect of beta-blockers (BBL) on lung function [10-16]. It is well established that BBL, even relatively cardioselective agents, can produce bronchoconstriction and thereby worsen respiratory flows and symptoms in patients with asthma or chronic obstructive pulmonary disease (COPD) [10,16,12]. However, two recent studies suggested that the treatment of cardiovascular diseases with cardioselective BBL, may reduce morbidity and mortality in patients with COPD [11,15]. Two systematic reviews of randomized controlled trials showed that the use of cardioselective BBL in patients with asthma or COPD has no adverse effects on lung function or respiratory symptoms [13,14]. However, these studies investigated the potential effect of BBL intake on lung function mainly in patients with already existing pulmonary diseases. The association between blood pressure, antihypertensive drug treatment and limited lung function in a population-based setting is much less investigated. Thus, the aim of this analysis is to determine whether hypertension as well as antihypertensive medication has an adverse effect on lung function in a general adult population.

## Methods

### Study population

The KORA F4 study is a follow-up of the KORA S4 study, a population-based health survey conducted in the city of Augsburg and two surrounding counties between 1999 and 2001. A total sample of 6640 subjects was drawn from the target population consisting of all German residents of the region aged 25 to 74 years.

Of all 4261 participants of the S4 baseline study, 3080 also participated in the 7-year follow-up F4 study. Persons were considered ineligible for F4 if they had died in the meantime (n = 176, 4%), lived outside the study region or were completely lost to follow-up (n = 206, 5%), or had demanded deletion of their address data (n = 12, 0.2%). Of the remaining 3867 eligible persons, 174 could not be contacted, 218 were unable to come because they were too ill or had no time, and 395 were not willing to participate in this follow-up, giving a response rate of 79.6%. Our study focuses on a subset of 1319 persons aged 40-65 years, because only this age-restricted subset performed both blood pressure measurements and lung function tests. The clinical examinations and interviews were performed at the same day. Overall, the KORA F4 study was conducted between 2006 and 2008.

The investigations were carried out in accordance with the Declaration of Helsinki, including written informed consent of all participants. All study methods were approved by the ethics committee of the Bavarian Chamber of Physicians, Munich.

### Outcome assessment

#### Lung function

Lung function examinations, i.e. spirometry, were conducted based on the American Thoracic Society (ATS) criteria [17] and the recommendations of the European Community for Steel and Coal (ECCS) [18]. The participants performed at least three forced expiratory lung function manoeuvres in order to obtain a minimum of two acceptable and reproducible values. Before the tests the examiner demonstrated the correct performance of the manoeuvres and then the individuals were supervised throughout the tests. According to the ATS recommendations [17] the tests were performed in a sitting position and with wearing noseclips. The best results for FVC and FEV_1 _were taken and percent predicted values were calculated according Quanjer et al. [18].

#### Blood pressure, medication and other determinats

Blood pressure was measured using a validated automatic device (OMRON HEM 705-CP). Three independent blood pressure measurements were taken with a 3-minute pause after a rest of at least 5 minutes in a sitting position on the right arm. The mean of the last two measurements was used for the current analyses. High blood pressure (HBP) was defined as blood pressure ≥ 140 mm Hg systolic or 90 mm Hg diastolic (with or without antihypertensive medications). Additionally, anthropometric measurements, computer-assisted standardized interviews and self-administered questionnaires on lifestyle and health related factors, medical history and respiratory symptoms were performed. Cardiovascular (heart attack, stroke) and pulmonary diseases (asthma, chronic bronchitis) were based on self-reported physician's diagnosis. The smoking status (current, former, or never-smokers) was assessed by self-report. Education level was defined by the highest graduation (less than O-level, O-level and more than O-level). Furthermore, the use of medication within the last seven days before the examination was ascertained by an instrument for database-assisted online collection of medication data (IDOM) [19]. The following substance classes were considered as antihypertensive medication according to the recommendations of the German Hypertension Association [20]: Antihypertensives (ATC code C02), diuretics (ATC code C03), beta-blocker (ATC code C07), calcium antagonists (ATC code C08), ACE inhibitors and angiotensin antagonists (ATC code C09). Finally, the following classes of high blood pressure based on the blood pressure measurement (HBP ≥ 140/90 mmHg) and antihypertensive medication were defined:

- A. HBP: high blood pressure regardless of its medical treatment

- B. HBP or medication: high blood pressure or the use of antihypertensive medication

- C. HBP and medication: high blood pressure and the use of antihypertensive medication; treated but uncontrolled hypertension

- D. Only HBP: high blood pressure, but no antihypertensive medication; untreated hypertension

- E. Only medication for HBP: antihypertensive medication, but no high blood pressure; treated and controlled hypertension

- F. Medication for HBP: antihypertensive medication independent of high blood pressure

### Statistical analyses

Descriptive analysis for the study population, blood pressure and lung function measures was done using chi-square and Kruskal Wallis tests to determine significance levels. Kruskal-Wallis tests and multivariable linear regression models were applied to study the association between the abovementioned classes of high blood pressure and antihypertensive medication and lung function outcomes. Additionally, high blood pressure and antihypertensive medication were used as individual variables in one regression model. P-values < 0.05 were considered statistically significant for all analyses. All statistical analysis was performed with SAS, version 9.13.

## Results

Table 1 shows gender-specific characteristics of the 1319 participants with complete data on lung function tests and blood pressure measurements. Women had a significantly lower body mass index (BMI), smoked less and their blood pressure readings and absolute lung function values were lower, whereas their percent predicted lung function values were higher compared to men (p < 0.01 for each comparison). Overall high blood pressure was less prevalent in women compared to men (10.3% versus 23.1%; p < 0.01). However, for the use of antihypertensive medication there was no difference between women and men.

**Table 1:** Characteristics of the study population based on KORA F4, persons aged 40-65 years with blood pressure measurements and lung function tests

	Men (N = 618)	Women (N = 701)	
	Mean ± SD	Mean ± SD	**P-Value**^**#**^
Age (years)	51.6 ± 5.8	51.5 ± 5.6	0.83
BMI (kg/m^2^)	28.0 ± 4.5	26.9 ± 5.2	<0.01
Packyears (y)	21.8 ± 20.1	13.4 ± 12.9	<0.01
Blood pressure			
Mean SBP (mm Hg)	126.3 ± 16.1	114.9 ± 15.8	<0.01
Mean DBP (mm Hg)	80.1 ± 9.8	74.1 ± 9.0	<0.01
Lung function			
FEV_1 _(l)	3.9 ± 0.7	2.8 ± 0.5	<0.01
FVC (l)	5.0 ± 0.8	3.6 ± 0.6	<0.01
FEV_1 _% predicted^**$**^	107.2 ± 16.2	110.1 ± 17.2	<0.01
FVC % predicted^**$**^	111.5 ± 13.7	120.2 ± 16.0	<0.01

	**n (%)**	**n (%)**	**P-Value**^**#**^

Pulmonary and cardiac diseases			
Asthma	50 (8.1)	69 (9.8)	0.27
Chronic bronchitis	49 (8.0)	58 (8.3)	0.81
Heart attack	17 (2.8)	11 (1.6)	0.14
Stroke	15 (2.4)	2 (0.3)	<0.01
Smoking status			
current	155 (25.1)	150 (21.4)	0.11
former	268 (43.4)	236 (33.7)	<0.01
never	195 (31.6)	315 (45.0)	<0.01
High blood pressure and medication*classes			
HBP	143 (23.1)	72 (10.3)	<0.01
HBP or medication*	221 (35.8)	177 (25.3)	<0.01
HBP and medication*	46 (7.4)	22 (3.1)	<0.01
Only HBP	97 (15.7)	50 (7.1)	<0.01
Only medication*for HBP	78 (12.6)	105 (15.0)	0.22
Medication* for HBP	124 (20.1)	127 (18.1)	0.37
Beta-blocker	68 (11.4)	82 (11.8)	0.67
ACE inhibitor	52 (8.4)	44 (6.3)	0.14
Angiotensin antagonists	41 (6.6)	33 (4.7)	0.13
Diuretics	63 (10.2)	60 (8.6)	0.32
Calcium antagonists	25 (4.1)	25 (3.6)	0.65
Other antihypertensive drugs	3 (0.5)	4 (0.6)	0.83

SBP: systolic blood pressure; DBP: diastolic blood pressure; HBP: high blood pressure

(≥140/90 mmHg); FEV_1_: forced expiratory volume in one second; FVC: forced vital capacity;

* Medication: antihypertensive medication; ^$ ^% predicted values according to Quanjer; ^# ^Obtained from the chi-square test when comparing frequencies and from the Kruskal Wallis test when comparing mean values.

In subjects with high blood pressure FEV_1 _% predicted (105.8 ± 16.0 versus 109.3 ± 16.9; p < 0.01) and FVC % predicted (111.6 ± 14.8 versus 117.0 ± 15.5; p < 0.01) were significant lower (Table 2). A similar significant difference of FEV_1 _% predicted (105.4 ± 16.4 versus 109.5 ± 16.8; p < 0.01) and of FVC % predicted (111.9 ± 16.1 versus 117.1 ± 15.3; p < 0.01) could be shown for the use of antihypertensive medication irrespective of high blood pressure. Furthermore, an effective blood pressure treatment and an ineffective blood pressure treatment, meaning high blood pressure despite the use of antihypertensive medication, were associated with lower FEV_1 _% (p = 0.04 and p < 0.01, respectively) and FVC % predicted values (p = 0.01 and p < 0.01, respectively).

**Table 2:** Crude association between high blood pressure, antihypertensive medication and lung function

	N	**FEV**_**1 **_**%**^**$**^	**P-Value**^**#**^	**FVC %**^**$**^	**P-Value**^**#**^
HBP					
Yes	215	105.8 ± 16.0	<0.01	111.6 ± 14.8	<0.01
No	1104	109.3 ± 16.9		117.0 ± 15.5	
HBP or medication*					
Yes	398	106.0 ± 16.3	<0.01	112.3 ± 15.4	<0.01
No	921	109.9 ± 16.9		117.8 ± 15.3	
HBP and medication*					
Yes	68	103.1 ± 15.7	<0.01	108.5 ± 15.8	<0.01
No	1251	109.1 ± 16.8		116.6 ± 15.4	
Only HBP					
Yes	147	107.0 ± 16.0	0.19	112.9 ± 14.2	0.01
No	1172	109.0 ± 16.9		116.5 ± 15.7	
Only medication* for HBP					
Yes	183	106.3 ± 16.7	0.04	113.2 ± 16.0	0.01
No	1136	109.2 ± 16.8		116.6 ± 15.4	
Medication* for HBP					
Yes	251	105.4 ± 16.4	<0.01	111.9 ± 16.1	<0.01
No	1068	109.5 ± 16.8		117.1 ± 15.3	
Medication* for HBP					
Beta-blocker	150	105.8 ± 16.1	0.01	112.0 ± 15.7	<0.01
Other antihypertensive drugs	101	104.9 ± 16.9	0.01	111.7 ± 16.7	<0.01
No antihypertensive drugs	1068	109.5 ± 16.8		117.1 ± 15.3	

HBP: high blood pressure (≥140/90 mmHg); FEV_1_: forced expiratory volume in one second; FVC: forced vital capacity; * Medication: antihypertensive medication; ^$ ^% predicted values according to Quanjer: mean ± standard deviation; ^# ^Obtained from Kruskal Wallis Test.

Similar results for the association between high blood pressure, antihypertensive medication and lung function could be shown in men. In women FEV_1 _and FVC % predicted values did not differ between subjects with and without high blood pressure. However, the use of antihypertensive medication irrespective of high blood pressure was associated with a significant reduced FEV_1 _and FVC % values in women (p = 0.02 and p < 0.01, respectively).

The descriptive analysis (Table 2) of the association between lung function and antihypertensive medication showed that both BBL and other antihypertensive medication, as for example ACE inhibitors, angiotensin antagonists, diuretics or calcium antagonist, are associated with reduced FEV_1 _and FVC % predicted values (p = 0.01 and p < 0.01, respectively).

The application of multivariable regression models revealed that high blood pressure as well as antihypertensive medication are associated with lower FEV_1 _(p = 0.02 and p = 0.05, respectively) and FVC values (p = 0.01 and p = 0.05, respectively) after adjusting for sex, age, height, weight, education level, packyears of smoking, pulmonary and cardiac diseases (Table 3, Model 1 and 3). When using both high blood pressure and antihypertensive medication as individual variables in one regression model, it could be shown, that both variables were associated with reduced lung function values (Model 6). However, antihypertensive medication showed only a trend toward a significant association with lower FEV_1 _and FVC values (each p = 0.08). Furthermore, the adjusted regression models with mutual exclusive categories pointed out that the combination of high blood pressure and the use of antihypertensive medication had the strongest negative effect on lung function (Model 4). Thus, among treated but not controlled hypertensive subjects FEV_1 _had a lower volume of 160 mL and FVC of 170 mL compared to subjects with no high blood pressure and no antihypertensive medication (each p = 0.02 and p = 0.02). A detailed analysis of antihypertensive medication showed that the use of BBL was associated with reduced FEV_1 _and FVC values, whereas other antihypertensive medication had no effect on lung function (Model 5). However, it has to be considered that the effect of BBL was significant for FVC (p = 0.03) while for FEV_1 _the association was of borderline significance (p = 0.07). A further model including BBL, other antihypertensive medication and high blood pressure showed similar negative effects of BBL on FVC (p = 0.04) and FEV_1 _(p = 0.07). Besides, high blood pressure was associated with reduced FVC (p = 0.01) and FEV_1 _(p = 0.03) values, too (Model 5a). An additional sensitivity analysis of the models 5 and 6, where we excluded subjects with obstructive lung diseases, showed that the effect of BBL still exists. Although the significance level declined the magnitude effect estimates did not change. For all multivariable regression models the adjusted r-squared value was 0.65 for FEV_1 _and 0.73 for FVC.

**Table 3:** Association between high blood pressure, antihypertensive medication and lung function - results of the multivariable regression analysis

**Models for FEV**_**1 **_**(L)**					Models for FVC (L)			
**Nr**.	Variable	Estimate	SD	**P **^**#**^	Variable	Estimate	SD	**P **^**#**^
	*HBP and antihypertensive medication classes*				*HBP and antihypertensive medication classes*			
1.	HBP	-0.09	0.04	0.02	HBP	-0.11	0.04	0.01
2.	HBP or medication	-0.08	0.03	0.01	HBP or medication	-0.10	0.03	<0.01
3.	Medication	-0.07	0.04	0.05	Medication	-0.08	0.04	0.05
	*Models with mutual exclusive categories*				*Models with mutual exclusive categories*			
4.	No HBP, no medication^§^				No HBP, no medication^§^			
	HBP and medication	-0.16	0.06	0.02	HBP and medication	-0.17	0.07	0.02
	Only HBP	-0.08	0.04	0.09	Only HBP	-0.10	0.05	0.04
	Only medication	-0.06	0.04	0.14	Only medication	-0.07	0.05	0.12
5.	No anti-hypertensive drugs^§^				No anti-hypertensive drugs^§^			
	Beta blocker	-0.08	0.05	0.07	Beta blocker	-0.11	0.05	0.03
	Other anti-hypertensive drugs	-0.06	0.05	0.28	Other anti-hypertensive drugs	-0.04	0.06	0.51
5a.	No anti-hypertensive drugs^§^				No anti-hypertensive drugs^§^			
	Beta blocker	-0.08	0.05	0.07	Beta blocker	-0.10	0.05	0.04
	Other anti-hypertensive drugs	-0.04	0.05	0.41	Other anti-hypertensive drugs	-0.02	0.06	0.72
	HBP	-0.08	0.04	0.03	HBP	-0.10	0.04	0.01
	*HBP and antihypertensive medication as individual variables*				*HBP and antihypertensive medication as individual variables*			
6.	HBP	-0.08	0.04	0.03	HBP	-0.10	0.04	0.02
	Medication	-0.07	0.04	0.08	Medication	-0.07	0.04	0.08

Multivariable regression analysis using the high blood pressure and antihypertensive medication classes (Model 1-3), mutual exclusive categories (Model 4,5,5a) and high blood pressure and antihypertensive medication as individual variables (Model 6).

SD: standard deviation; FEV_1_: forced expiratory volume in one second; FVC: forced vital capacity; HBP: high blood pressure (≥140/90 mmHg); Medication: antihypertensive medication; ^§ ^Reference category; ^#^P-Value: all models are adjusted for gender, age, height, weight, education level, packyears, pulmonary and cardiac diseases.

A further sensitivity analysis regarding the possible effect modification by gender showed no gender difference for FEV_1 _for all models. The multivariable regression models for FVC, however, showed a significant interaction between gender and high blood pressure, indicating that high blood pressure has a lower effect on lung function in women compared to men. Further analyses regarding the ratio FEV_1_/FVC showed no significant association between high blood pressure or antihypertensive medication and the ratio FEV_1_/FVC.

## Discussion

The present analysis of a population-based study demonstrates that both high blood pressure and the use of BBL are associated with reduced lung function, whereas other antihypertensive medications have no effect on lung function.

Our findings are in line with previous observations that blood pressure and lung function are inversely associated [5,6,9]. But most of these studies did not differentiate between the effect of high blood pressure and the effect of antihypertensive medication on lung function. Instead they defined hypertension as elevated blood pressure or use of antihypertensive medication. One study, however, found no difference in FEV_1 _and FVC between hypertensive subjects that used or did not use beta blocking antihypertensives [6], but they did not specifically address the effect of antihypertensive medication independent of high blood pressure on lung function.

Thus, our study might substantially add to the question, whether antihypertensive BBL medication independent of high blood pressure has adverse effects on lung function. Beta-adrenergic receptors (β-ARs) play a key role in the regulation of bronchomotor tone [21]. In the respiratory system most of the β-ARs are β2-ARs. However, there are β1-ARs, too, which are responsible for the respiratory effects of cardioselective β1-antagonists. Two systematic reviews suggest that cardioselective BBL do not produce adverse respiratory effects in patients with asthma or COPD [13,14]. These randomized clinical trials examined only patients with already existing pulmonary diseases and not healthy subjects. Other studies provide evidence that BBL medication, even relatively cardioselective agents, produce bronchoconstriction and thereby worsen respiratory flows in asthmatic patients [10,16]. Our results indicate that the use of BBL medication is associated with a slight reduction of FEV_1 _and FVC. Interestingly, the FEV_1_/FVC ratio was found not to be affected by BBL medication suggesting that the expired volume, FVC, is lowered in proportion. Indeed, the drug-specific effect of BBL medication is more pronounced on FVC than on FEV_1_. This supports the hypothesis that not airway obstruction, but rather restriction is the more likely mechanism involved in the effect of BBL medication on lung function. For instance, possible effects on the respiratory muscle strength have to be considered. It is well established that beta agonists improve the performance of skeletal muscles [22] and also positively affect respiratory muscle strength [23,24]. The opposite effect by BBL medication is suggested by a recent study from Frankenstein et al. performed in patients with chronic heart failure [25]. Thus, we hypothesize that BBL medication may result in a slight reduction of expiratory muscle strength causing a proportional decrease of FEV_1 _and FVC. However, further studies directly addressing this issue are required.

When reviewing our results, it becomes apparent that from a statistical point of view both high blood pressure and antihypertensive BBL medication have an effect on lung function measurements. But the observed lung function differences between exposed und non-exposed subjects are relatively small, meaning that they have no direct clinical consequence in healthy individuals. However, we could show that among treated but not controlled hypertensive subjects FEV_1 _had a lower volume of 160 mL compared to subjects with no high blood pressure and no antihypertensive medication. This finding might be of importance on the population level. One possible explanation for this significant lung function reduction might be an additive effect of both treatment and persistent high blood pressure. However, the cross-sectional study design makes it difficult to disentangle the effects of high blood pressure and antihypertensive medication. Thus, it allows only statements about a single point in time and does not allow evaluating the effect of long-standing high blood pressure. Another explanation for this lung function decrement could be that those with persistent hypertension despite medical treatment have a higher underlying blood pressure compared to effectively treated subject. Moreover, the effects of high blood pressure and antihypertensive medication are highly correlated. A detailed analysis of antihypertensive medication indicates that BBL medication and not any other antihypertensive medication is associated with a reduced lung function. This negative effect of BBL medication still remains, when BBL, other antihypertensive medication and high blood pressure are analysed in the same model. Besides, BBL are the most common prescribed antihypertensive medication and it has to be considered that BBL medication might be prescribed for other indications than hypertension, as for example, coronary heart diseases or heart failure, too. This again suggests that the effect of antihypertensive BBL medication on lung function is mainly ascribed to the medicament and not to the indication. Furthermore, a variety of confounders might affect the association between high blood pressure, antihypertensive medication and lung function. Cigarette smoking is a common risk factor for both impaired lung function and high blood pressure and BMI might have an effect on lung function. However, adjustment for these possible confounders did not influence our results. Moreover, we could show that the association was not affected by the concomitance of pulmonary diseases and that the negative effect of BBL medication on lung function is not modified by obstructive lung diseases. This supports our interpretation that BBL have an effect on lung function in the general population. Besides, our results suggest that there may be an effect modification by gender. We could show that in women the percent predicted lung function values did not differ between subjects with and without high blood pressure. Furthermore, the multivariable regression analysis revealed a significant interaction between gender and high blood pressure. This might possibly indicate that high blood pressure has minor effect on lung function in women compared to men.

The large sample size and the population-based setting are a major strength of this study. Furthermore, it is one of few investigations differentiating between the effect of blood pressure and antihypertensive drug treatment on lung function. Nevertheless, this study has some possible limitation. Methodological bias might lead to insufficient lung function measurements. Thus in patients with high blood pressure lung function tests might be stopped earlier, because the respiratory effort might cause an increase in blood pressure. However, we consider this possible bias unlikely to affect our findings. Besides, selection bias might limit the representative status of the population sample included in our analysis. We had to restrict our analysis to subjects aged 40-65 years, because only this age-restricted subset performed lung function tests. However, as this was a random sample, we consider that our population sample is highly representative for this age group of the Augsburg population. Moreover, the cross-sectional study design makes it difficult to make a clear statement about the temporal sequence and causality between high blood pressure, its treatment and lung function. Several prospective studies indicated that high blood pressure is a risk factor for reduced lung function as well as impaired lung function increases the risk for the development of high blood pressure [7-9,26]. Besides, one has to consider the possible effect of long-standing high blood pressure. For example, subjects with currently normal blood pressure under medication might have had high blood pressure for a long time before it was recognized and treated. Therefore, it is necessary to evaluate the temporal sequence, acute and chronic effects and the causality between high blood pressure, its medical treatment and lung function in further prospective studies.

## Conclusions

Our findings are in line with previous observations showing an inverse association between blood pressure and lung function. Furthermore, our analysis indicates that BBL medication and not any other antihypertensive treatment is associated with reduced lung function in a general adult population. Thus, our findings may serve as a basis for experimental testing, as for example by adding measurement of respiratory functions to outcomes of 'hypertensive' trials.

## Competing interests

The authors declare that they have no competing interests.

## Authors' contributions

ES was responsible for the data analysis, interpretation of data and manuscript preparation. JH and ES developed the statistical analysis plan. SK, HS, SG, CM, MH, AP, H-EW, JB, RMH and JH assisted in the interpretation and critical revision of the results. SK, HS, CM, MH, H-EW and AP were responsible for the data. All authors read and approved the final manuscript.
